# Higher white-nose syndrome fungal isolate yields from UV-guided wing biopsies compared with skin swabs and optimal culture media

**DOI:** 10.1186/s12917-023-03603-6

**Published:** 2023-02-10

**Authors:** Veronika Seidlova, Jiri Pikula, Miroslav Kolarik, Alena Nováková, Adela Cmokova, Astghik Ghazaryan, Monika Nemcova, Sarka Bednarikova, Sneha Patra, Tomasz Kokurewicz, Vladimir Piacek, Jan Zukal

**Affiliations:** 1 Department of Ecology and Diseases of Zoo Animals, Game, Fish and Bees, University of Veterinary Sciences Brno, Palackého tř. 1946/1, 612 42 Brno, Czech Republic; 2 Institute of Vertebrate Biology, Czech Academy of Sciences, Květná 8, 603 65 Brno, Czech Republic; 3 Laboratory of Fungal Genetics and Metabolism, Institute of Microbiology, Czech Academy of Sciences, Vídeňská, 1083, 142 20 Prague 4, Czech Republic; 4 Department of Zoology, Yerevan State University, 1 Alex Manoogian, 0025 Yerevan, Armenia; 5 CzechGlobe, Global Change Research Institute of the Czech Academy of Sciences, Bělidla 986/4a, 603 00 Brno, Czech Republic; 6 Department of Vertebrate Ecology and Palaeontology, Institute of Environmental Biology, Wrocław University of Environmental and Life Sciences, Kożuchowska 5B, 51-631 Wrocław, Poland; 7 Department of Botany and Zoology, Masaryk University, Kotlářská 267/2, 611 37 Brno, Czech Republic

**Keywords:** Bats, UV lesions, Fungal infection, Fungal culture, *Pseudogymnoascus destructans* skin microbiota

## Abstract

**Background:**

North American bat populations have suffered severe declines over the last decade due to the *Pseudogymnoascus destructans* fungus infection. The skin disease associated with this causative agent, known as white-nose syndrome (WNS), is specific to bats hibernating in temperate regions. As cultured fungal isolates are required for epidemiological and phylogeographical studies, the purpose of the present work was to compare the efficacy and reliability of different culture approaches based on either skin swabs or wing membrane tissue biopsies for obtaining viable fungal isolates of *P. destructans.*

**Results:**

In total, we collected and analysed 69 fungal and 65 bacterial skin swabs and 51 wing membrane tissue biopsies from three bat species in the Czech Republic, Poland and the Republic of Armenia. From these, we obtained 12 viable *P. destructans* culture isolates*.*

**Conclusions:**

Our results indicated that the efficacy of cultures based on wing membrane biopsies were significantly higher. Cultivable samples tended to be based on collections from bats with lower body surface temperature and higher counts of UV-visualised lesions. While cultures based on both skin swabs and wing membrane tissue biopsies can be utilised for monitoring and surveillance of *P. destructans* in bat populations, wing membrane biopsies guided by UV light for skin lesions proved higher efficacy. Interactions between bacteria on the host's skin also appear to play an important role.

## Background

Underground environments are unique as, on the one hand they are generally considered to be relatively nutrient-poor ecosystems [1], while on the other the high microbial diversity of caves indicates that each has its own characteristics [2, 3]. The limited resources of subterranean spaces have led microorganisms to specific adaptations, resulting in either cooperation or competition for inadequate energy and nutrient resources [4]. Fungi in particular show high ecological plasticity, adopting a range of different lifestyles such as saprophytism, parasitism or symbiosis [5, 6].

*Pseudogymnoascus destructans* is a slow growing, psychrophilic fungus that utilises saprophytic growth underground [7–9] or pathogenic growth on hibernating bats [10]. As the causative agent of white-nose syndrome (WNS), *P. destructans* has been responsible for the most devastating infectious outbreak in wild mammals yet recorded over an extensive area of the Nearctic region [11–13]. Fungal growths of *P. destructans* are predominantly found on the auricular, nasal and facial skin of hibernating bats; however, the most clinically severe lesions affect the patagial membranes [10]. In the past, WNS diagnosis required euthanasia and/or disease-associated death of bats for histopathology analysis to identify pathognomonic cupping erosions in the skin samples [14]. More recently, UV light detection of lesions in bat wings has been validated as a non-invasive method comparable in sensitivity with histopathology [15]. This non-lethal and field-applicable method is not only useful for screening hibernating bats for the WNS disease, but also for targeting wing biopsies over yellow-to-orange fluorescing skin lesions.

Epidemiological and phylogeographic studies of WNS require cultured fungal isolates [16–20]. Likewise, investigations of growth characteristics of the agent, its metabolic activity, production and toxicity of secondary metabolites, sensitivity to antimycotics and other in vitro experiments would not be possible without obtaining such isolates [21–27]. There are essentially two approaches for collecting samples for fungal culture from the bats in the field, i.e. a skin surface swab targeting visible fungal growths or a wing membrane tissue biopsy targeting skin lesions produced by the fungus. These two approaches can also be combined to maximise likelihood of obtaining a viable fungus suitable for culture in the laboratory.

To date, however, there have been no studies comparing the efficiency of skin swabs against wing biopsies for the establishment of viable *P. destructans* cultures. Here we describe an experimental study of WNS-affected bats, comparing fungal isolate yields from UV-guided wing biopsies and skin swabs. We predicted that tissue biopsies containing densely packed fungal hyphae will provide higher numbers of cultivable fungal units (both hyphae and conidia) compared with the skin surface swab. Tissue biopsies may also provide some protection and supply nutrients, allowing survival of the fungus during sample transport to the laboratory. Some microorganisms are known to inhibit growth of *P. destructans* [28, 29], hence bacterial contamination of samples may also influence the yield of fungal cultures. As such, we also examined the degree of interference between cultivable microbiota present on the skin and the yield of a viable fungal culture. Finally, we tested the suitability of different culture media for growing *P. destructans*.

## Material and methods

### Sample collection

For this experiment, we screened the complexity of skin bacterial and fungal microbiota of three bat species: the greater mouse-eared bat (*Myotis myotis*)*,* the lesser mouse-eared bat (*Myotis blythii*) and the greater horseshoe bat (*Rhinolophus ferrumequinum*)*,* in *P. destructans* contaminated caves and artificial underground shelters in the Czech Republic, Poland and the Republic of Armenia over two hibernation periods (each locality was visited just one time) covering January to April 2018 and 2019 (Table 1). In all cases, the bats were handled in such a way as to minimise stress and the duration of sampling procedures and all were released at the site after sampling was completed. Prior to handling, the animal’s surface body temperature was measured using a Raynger MX2 non-contact IR thermometer (Raytek Corporation, Santa Cruz, USA). A total of 82 bats were sampled in the study, with two or three sample types being collected from each bat. First, skin surface swabs were collected separetely for bacterial (right wing) and fungal cultivation (left wing). Second, a 4 mm skin biopsy from skin lesions found on wing membranes were obtained from each tested animal. Biopsy sites were chosen by trans-illuminating wing membranes with a 368 nm wavelength UV lamp, allowing identification of presumptive WNS lesions by yellow to orange fluorescence [15]. At the same time, a photograph was taken of the trans-illuminated wing, allowing the fluorescent areas to be manually enumerated in the laboratory, using the counting tool in the ImageJ software package [30].

**Table 1:** Number of individual bats sampled at each study site

**Country**	**Locality**	**Species**	**Year of sampling**	**Biopsy only**	**Swab only**	**Both samples**	**Total**	**UV positive**	**Bacterial swabs**
Czech Republic	Mine Velká Amerika	*Myotis myotis*	2018	-	2	-	2	1	-
	Sloupsko-Sosuvske Caves	*Myotis myotis*	2018	7	5	-	12	6	-
	Šimon and Juda mines	*Myotis myotis*	2019	3	2	9	14	14	-
Poland	Nietoperek bunker	*Myotis myotis*	2019	2	-	15	17	17	-
Armenia	Magel cave	*Rhinolophus ferrumequinum*	2019	-	17	-	17	4	20
	Mine Shikahogh	*Myotis blythii*	2019	1	5	14	20	20	20
	**Total**			**13**	**31**	**38**	**82**	**62**	**40**

### Laboratory isolation & culture of samples collected in the field

#### Bacterial swabs

Each bat from Armenia (*n* = 40) was individually sampled over the wing membrane surface using a sterile cotton, plastic-shafted swab and the sample in Amies medium (Copan Italia S.p.A, Brescia, Italy). The tubes were stored at 5–8 °C and processed within 5–10 days, depending on the geographic region where sample collection was performed and the time taken to travel back to the laboratory. Agar plates (Columbia Agar Base, Oxoid, Basingstoke, Hampshire, UK) and MacConkey agar (MCA; Oxoid, Basingstoke, Hamshire, UK) were used for routine isolation of bacteria. Two replicates of each sample were inoculated onto the agar plates, which were then incubated under aerobic conditions at 20 °C for three days for maximal growth of psychrophilic bacteria. After first characterising the bacterial isolates using MALDI-TOF mass spectroscopy, bacterial DNA were extracted using the commercial NucleoSpin® Microbial DNA extraction kit (Macherey-101 Nagel, Germany). For PCR amplification, we used the universal bacterial 16S rRNA gene primers 27F (5`-AGAGTTTGATCCTGGCTCAG-3`) and 1492R (5`-GGTTACCTTGTTACGACTT-3`). Each 25 μl reaction contained ca. 50 ng of genomic DNA, 12.5 μl Q5® High-Fidelity 2X Master Mix (New England BioLabs, UK), 2 μl nuclease free water (Bioron, Germany), 2 μl each of 10 mM forward and reverse primer, and 2 μl of sterile deionised water. The reaction conditions comprised an initial denaturation cycle of 95 °C for 15 min; followed by 35 cycles of denaturation for 1 min at 95 °C, primer annealing for 1 min at 58 °C and extension for 2 min at 72 °C, followed by a final extension for 10 min at 72 °C. PCR products were commercially sequenced using Sanger sequencing at SEQme Inc. (Czech Republic) using the universal primers 800R (5´-TACCAGGGTATCTAATCC-3´) and 1492R (5´-GGTTACCTTGTTACGACTT-3´). The sequences were aligned using the BioEdit Sequence Alignment Editor v.5.0.9 [31] and compared with known sequences in the NCBI database using BLAST (GenBank) to identify similar sequence alignments.

#### Fungal swabs

Fungal swabs were taken using sterile 15 cm swabs with a plastic applicator stored in transport tubes. The tubes were stored at 5–8 °C and processed within 5–10 days, depending on the geographic region where sample collection was performed and the time taken to travel back to the laboratory. At the laboratory, the swab was moistened in 0.1% sterile peptone water and rubbed onto glucose agar with chloramphenicol (GKCH) and cultivated at 10 °C for 14–30 days.

#### Skin biopsies

Wing punch biopsies of suspect fungal lesions were placed into sterile vials with 15 µl of NaCl 0.9% (for humidity), stored at 5–8 °C and, within 5–10 days (depending on the country of sampling), placed on Malt Extract Agar growth medium with ATB (chloramphenicol 50 mg/l) and cultivated at 10 °C for 14–30 days.

First, colony diameter and sporulation were compared on eight standardly used complex mycological media to find the best medium for routine use and to serve as a control. The following media were tested: glucose agar (GK; yeast extract 5 g/l, glucose 20 g/l, agar 15 g/l; pH = 6.1); yeast extract GK with chloramphenicol (GKCH; GK with 0.1 g/l of chloramphenicol; pH = 6.1); soil extract agar (SEA; 20 g agar, 1000 ml of soil extract; pH = 6.4) [32]; soil extract agar with glucose and rose bengal (SEGA; 20 g glucose, 1 g NaNO3, 1 g K2HPO4, 70 mg rose bengal, 1000 ml of soil extract; pH = 6.4) [33]; Sabouraud dextrose agar (SDA; 40 g/l glucose, 10 g/l pepton, 20 g/l agar; pH = 5.8) [34]; SDA with cycloheximide (SDAC; SDA with 0.1 g/l cycloheximide; pH = 5.8) [34]; potato carrot agar (PCA; 20 g/l potato; 20 g/l carrot, 20 g/l agar; pH = 6.4) [33]; and Czapek-Dox agar (CZ; 1 g K_2_HPO_4_, 10 ml CZ and 1 ml Cu–Zn concentrate, 5 g/l yeast extract, 30 g/l saccharose, 15 g/l agar; pH = 5.8) [34]. All test media were sterilised at 121 °C for 15 min and wrapped in Parafilm (Fisher Scientific, USA) after inoculation. The ten-strain set was then cultivated at 10 °C in darkness and measured after 7, 14, 21 and 28 days.

### Isolates and culture media; fungal optimal growth test

The ten *P. destructans* isolates (CCF 3938, 3941, 3943, 4103, 4124, 4126, 4128, 4129, 4131, 4132) used in this study were isolated between 2010 and 2011 from the muzzles and wings of *M. myotis* in the Czech Republic. The isolates, which were identified using morphological and molecular methods, are representative of both the mating types and genetic variability found in Eurasia [35, 36]. Experiments testing the suitability of culture media for growing *P. destructans* were performed on all isolates (ten-strain set) which were cultivated at 10 °C.

### Statistical analysis

Body surface temperature, number of UV lesions per bat, the number of bacterial species isolated from each bat and the *P. destructans* colony size (measured as a diameter in mm after one month of fungal cultivation at 10 °C) were tested for normality using the Kolmogorov–Smirnov and Shapiro–Wilk tests. As all parameters with exception of the colony size were non-normally distributed, even after transformation, they were then tested using the non-parametric Mann–Whitney U test. Difference between suitability of various microbiological culture media to obtain a *P. destructans* (fungal colony size) was tested using One-way ANOVA. The efficacy of each sampling method to yield a *P. destructans* culture isolate was assessed using the difference test between proportions. Due to the small sample size available for certain species, differences in fungal cultivation success between bat species was tested using the chi-squared test with Yates correction in Statistica for Windows® 13.2 (StatSoft, Inc., USA).

## Results

**Fig. 1:**
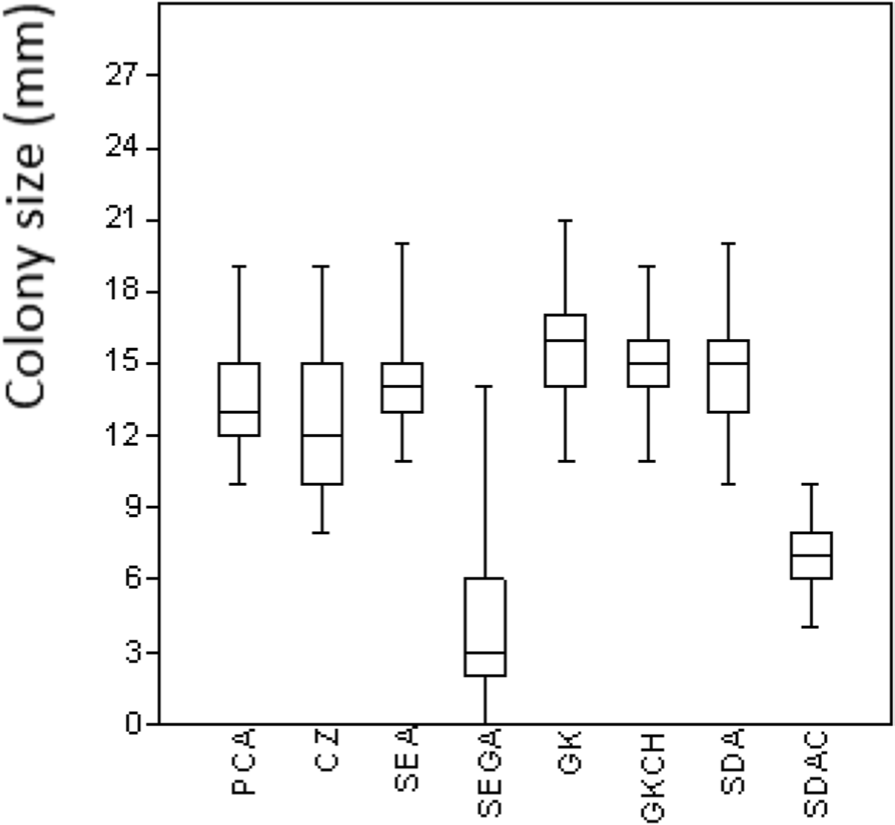
Relationship between *P. destructans* (ten strain set) colony size and cultivation medium after one month incubation at 10° C: potato carrot agar (PCA), Czapek agar (CZ), soil extract agar (SEA), soil extract agar with glucose and rose bengal (SEGA), yeast extract glucose agar (GK), yeast extract glucose agar with chloramphenicol (GKCH), sabouraud dextrose agar (SDA) and sabouraud dextrose agar with cycloheximide (SDAC) after one month of cultivation at 10 °C

Six of the eight standard test media (not SEGA or SDAC) proved to be suitable for growing *P. destructans* cultures (one-way ANOVA, *p* > 0.05; Fig. 1). The GK medium proved to be the best and cheapest variant and this was subsequently used for most of the experiments and for isolate storage.

**Fig. 2:**
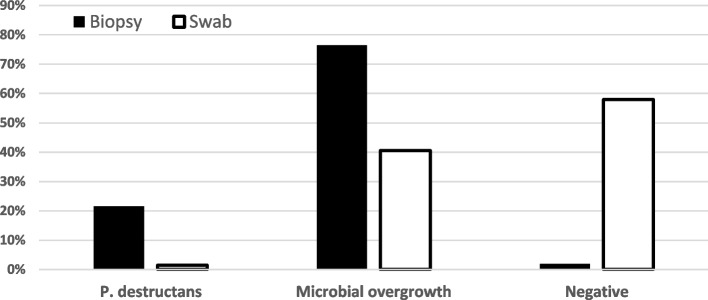
Proportion of samples yielding bacterial or fungal growth, including *P. destructans*. Results are based on wing membrane tissue biopsies of *Myotis blythii* (Armenia) guided by UV light for presumptive white-nose syndrome skin lesions in the plagiopatagium (biopsy) and non-targeted fungal skin surface swabs

In total, we obtained 12 viable culture isolates of the *P. destructans (Pd)* fungus (Fig. 2), with the efficacy of cultures based on wing membrane tissue biopsies (*n* = 11 biopsies with *Pd* isolates) being significantly higher (difference test between two proportions; *p* = 0.001) than fungal skin swabs (*n* = 1 swab with *Pd* isolate). The skin swab *Pd* isolate corresponded to *Pd* isolate obtained from a wing biopsy from the same bat. Microbial overgrowth was caused by one fungus, six filamentous molds and yeast species (Table 2). The fungus *Chaetomium* spp. was only found in swabs from the *M. myotis*. There was no significant difference in the number of cultures positive for *P. destructans,* obtained from *M. myotis* and *M. blythii* using the two methods of sampling (chi-square for swabs = 0.08; *p* = 0.778 and chi-square for wing biopsies = 0.3; *p* = 0.583). Likewise, there was no significant difference in the number of swabs, giving the positive results of culturing, between *Myotis-* and *Rhinolophus*-based samples (chi-square = 0.35; *p* = 0.553).

**Table 2:** Filamentous molds and yeasts causing microbial overgrowth of cultures. Abbreviations: Mbly – *Myotis blythii*, Mmyo – *Myotis myotis*, Rfer – *Rhinolophus ferrumequinum*

Identified fungi and yeasts	**Skin biopsy**			**Swab**		
	Mbly	Mmyo	Rfer	Mbly	Mmyo	Rfer
Hyaline filamentous ascomycete	+	+	not collected	+	+	+
Dematiaceous filamentous ascomycete	+	+		+	-	+
*Chaetomium* spp.	-	-		-	+	-
Yeasts	+	+		-	-	-
*Mucor* spp.	+	+		-	+	-
*Pseudogymnoascus pannorum* sl	+	+		+	+	+
*Penicillium* spp.	-	+		+	+	+
*Pseudogymnoascus destructans*	+	+		-	+	-
**Total number**	6	7	NC	4	6	4

**Fig. 3:**
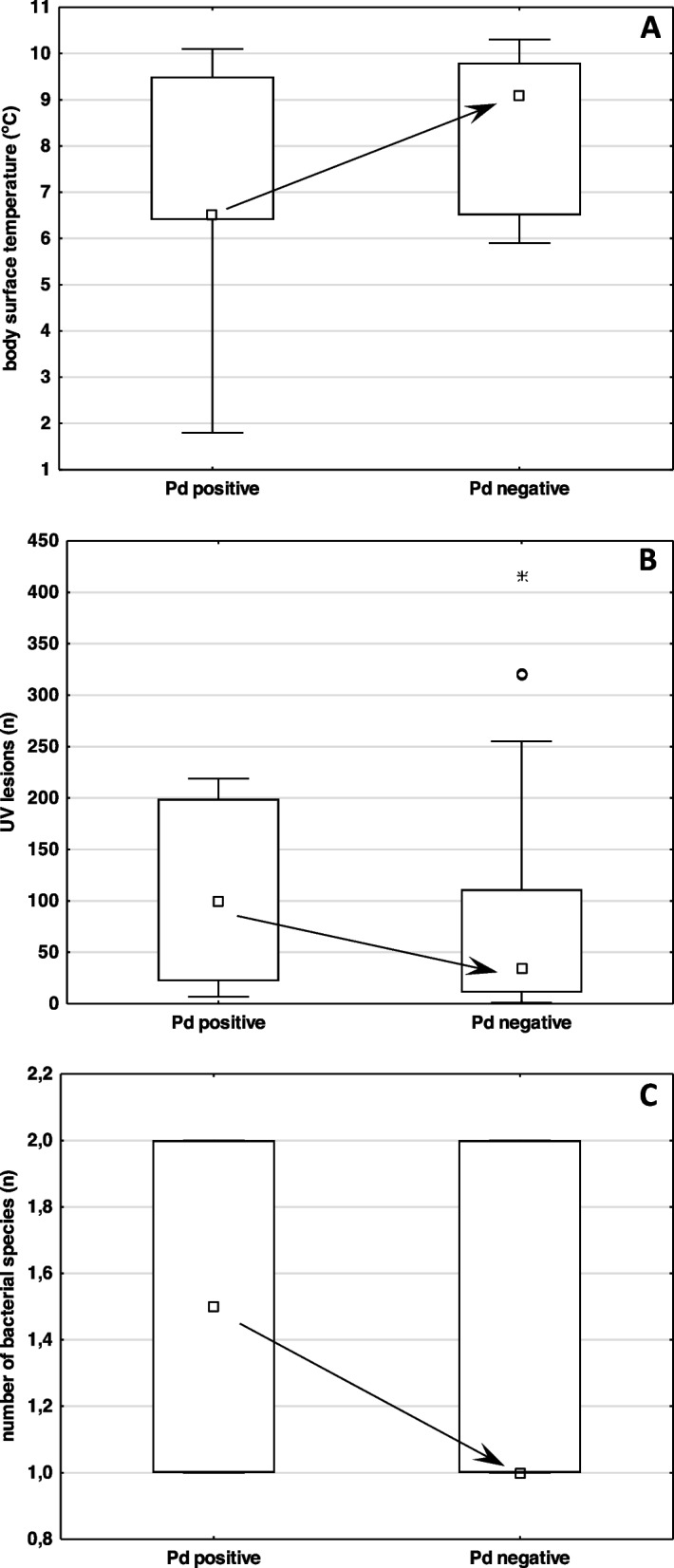
Factors influencing efficacy of wing membrane tissue biopsy sampling method. **A**—body surface temperature, **B**—the quantity of UV lesions per each bat, **C**—the number of bacterial species isolated from each bat. Arrows highlight insignificant trends of differences. Explanations: Pd – *Pseudogymnoascus destructans*, square – median, box – 25%-75%, whiskers – non-outlier range, dot – outliers and star – extremes

**Fig. 4:**
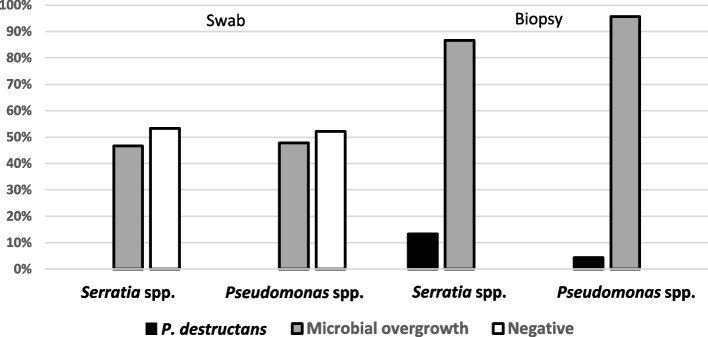
Differences between *Pseudogymnoascus destructans* cultivation yield with regard to the presence of two bacterial species (all samples from Armenia, *n* = 40). Results are based on wing membrane tissue biopsies guided by UV light for presumptive white-nose syndrome skin lesions in the patagium (biopsy) and skin surface swabs. Bacterial sample contamination representing the skin microbial community was determined as *Serratia* spp. and *Pseudomonas* spp

While cultivable samples from biopsies tended to be based on collections from bats with lower body surface temperature, higher UV-visualised lesion counts and a more diverse bacterial community (Fig. 3), all tests were non-significant (body surface temperature Z = -1.151; *p* = 0.250; number of UV-visualised skin lesions per bat Z = 0.735; *p* = 0.462; number of bacterial species isolated from each bat Z = 0.170; *p* = 0.865). Specifically, there was no difference in successful cultivations of *P. destructans* from wing membrane biopsies in the presence of *Serratia* spp. and/or *Pseudomonas* spp. bacteria (difference test between two proportions; *p* = 0.304; Fig. 4).

**Fig. 5:**
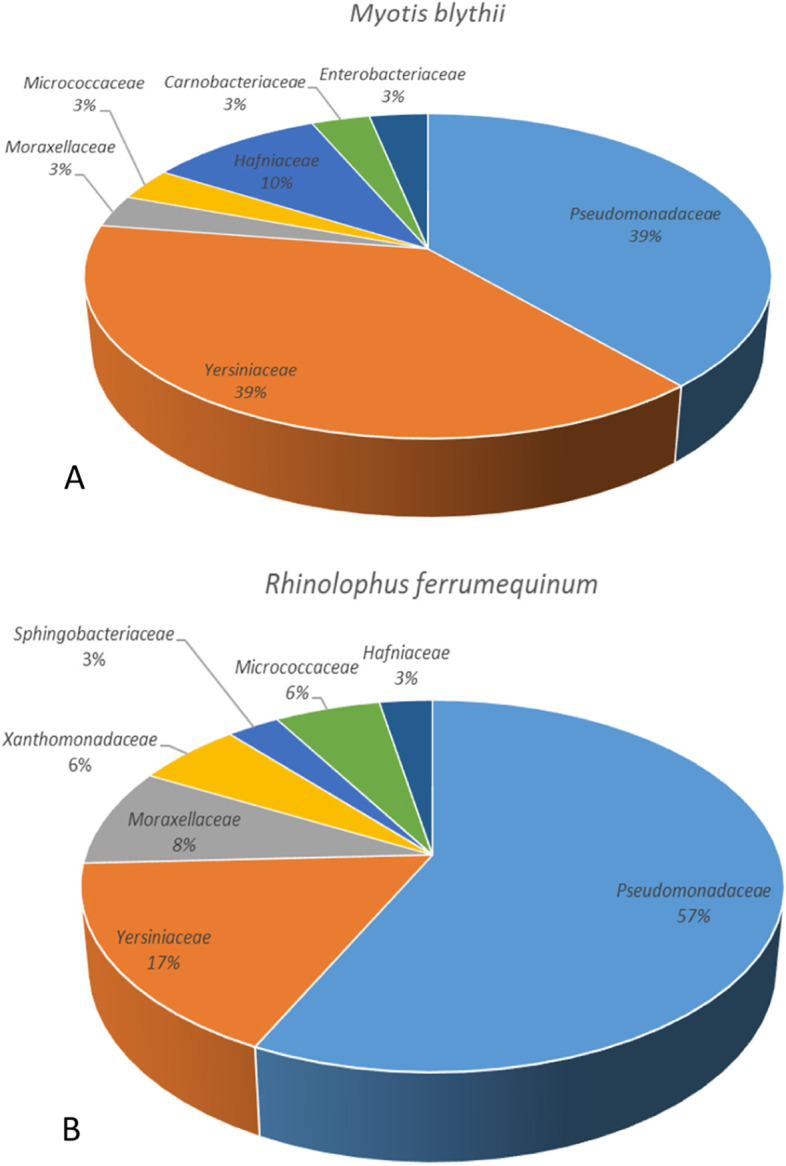
Percentage of bacterial taxons reflecting the average bacterial community diversity of Armenia bats, *Myotis blythii* (**A**; *n* = 20) and *Rhinolophus ferrumequinum* (**B**; *n* = 20). **A** Results are based on 16S rRNA sequence analysis. Gram negative: Moraxellaceae, Hafniaceae, Yersiniacea, Pseudomonadaceae, Enterobacteriaceae, Carnobacteriaceae; Gram positive: Carnobacteriaceae, Micrococcaceae. **B** Gram negative: Moraxellaceae, Xanthomonadaceae, Sphingobacteriaceae, Hafniaceae, Pseudomonadaceae; Gram positive: Micrococcaceae

Analysis of 16S rRNA identified seven dominant families in the skin microbiomes of *M. blythii* (Fig. 5, A) and *R. ferrumequinum* (Fig. 5, B) during late hibernation, with the ratio between gram-negative and gram-positive bacteria being 5:2 for *M. blythii* and 6:1 for *R. ferrumequinum*. Overall, both *R. ferrumequinum* and *M. blythii* bats displayed a similar diversity of bacterial species with some degree of bacterial species overlap including opportunistic and even zoonotic agents (Table 3).

**Table 3:** Bacterial diversity in *M. blythii* (*n* = 20) and *R. ferrumequinum* (*n* = 20) sampled in Armenia

***Rhinolophus ferrumequinum***	**Number of bacterial isolates**	***Myotis blythii***	**Number of bacteria isolates**
*Acinetobacter calcoaceticus*	3	*Acinetobacter guillouiae*	1
*Arthrobacter sp.*	2	*Arthrobacter sp.*	1
*Hafnia paralvei*	1	*Carnobacterium maltaromaticum*	1
*Pseudomonas azotoformans*	11	*Enterobacter cloacae*	1
*Pseudomonas fluorescens*	3	*Hafnia alvei*	2
*Pseudomonas gessardii*	1	*Hafnia paralvei*	1
*Pseudomonas sp.*	1	*Pseudomonas azotoformans*	4
*Serratia liquefaciens*	3	*Pseudomonas fluorescens*	3
*Serratia marcescens*	1	*Pseudomonas gessardii*	3
*Serratia proteamaculans*	2	*Pseudomonas spp.*	1
*Sphingobacterium sp.*	1	*Serratia liquefaciens*	10
*Stenotrophomonas maltophilia*	2	*Serratia proteamaculans*	3
		*Yersinia enterocolitica*^a^	1
**Total bacterial species**	12	**Total bacterial species**	13

^a^This bacterial isolate is considered zoonotic

## Discussion

The study and control of wildlife diseases is a challenging task. White-nose syndrome is an emerging infectious disease that can have devastating impacts on bat populations [37, 38]. Reliable sampling techniques are still needed to identify, isolate and characterise the fungus causing WNS. In this study, we were able to show that the ability to more readily obtain a viable *P. destructans* isolate differed depending on the technique used for sample collection from bats.

*P. destructans* infects the skin of its bat hosts [14], with pathology grades ranging from surface skin and/or hair follicle colonisation to epidermal cupping erosions, deep dermal and even full-thickness wing membrane invasion [39–41]. Understandably, the deeper the fungus invades the skin, the more difficult it becomes to detect, especially when non-invasive sampling techniques are used. In order to understand the pathology and progression of WNS in bats, samples are collected from bat cadavers or live hibernating bats using both non-invasive and invasive methods [42]. Use of non-lethal methods is especially important in the case of strictly protected European bat species and declining North American bat species, and is also advisable for bats being investigated for presence or absence of the WNS etiological agent in other temperate regions of the world [15, 36, 39].

The collection of clinical samples from bats with visible fungal growths using skin surface swabs is relatively straightforward, even under field conditions [43, 44]. However, infected bats may lose visible fungal growths as they clean themselves during arousal bouts. Likewise, the fungus may be wiped off a bat cadaver’s surface during transport to the laboratory. Skin swabs, therefore, may yield unreliable results in terms of obtaining a fungal culture isolate. A better alternative for the collection of samples for microbiological cultures may be skin biopsies, as the quantity of fungal elements within the tissue [45] allows the culture to continue growing from hyphae within the fungal biomass. While skin surface swabs probably contain higher amounts of conidia, it is still not known how the types of initial colony-forming units (i.e., conidia and/or hyphae) influence the likelihood of yielding a fungal isolate [42]. Other factors that may have impact on the culture yield include the intensity of infection, prevalence of WNS in the bat population under study, sample transport conditions, and growth characteristics, viability and persistence of the agent [11, 36, 42, 44, 46–49].

Compared with non-targeted skin swabbing, the diagnostic sensitivity of a culture yielding a *P. destructans* isolate is considerably increased when based on a UV-guided tissue biopsy (Fig. 2). The relatively low diagnostic sensitivity of fungal culture [40] from skin biopsies of about 20% necessitates a more sensitive diagnostic technique be combined to either confirm the presence of the fungal agent [50] or pathognomonic lesions it produces [14, 15, 39] when conducting surveillance for the pathogen or monitoring for WNS disease progression [48, 49, 51]. Real-time PCR is a highly sensitive and specific test, that can detect as few as 3.3 fg of genomic DNA extracted from *P. destructans* [50], while the diagnostic sensitivity and specificity of UV fluorescence for detecting WNS skin infection intensity can be up to 98.8% and 100%, respectively [15]. Standard WNS diagnosis outside the known geographic distribution of the infection requires finding pathognomonic histopathology [14] and detection of the *P. destuctans* agent either by qPCR [50] or by fungal culture [43]. However, while PCR- and histopathology-based methods can provide good data on infection intensity, PCR cannot distinguish between viable and non-viable *P. destructans* fungi at the time of collection, making it a poor indicator for the cultivable condition of the sample. On the other hand, histopathologic evidence of fungal skin invasion is a reliable indicator that the fungus was alive at the time of sample collection from a live bat. This does not, however, necessarily mean that the fungus will grow in culture.

A further problem is that overgrowth with competing microorganisms can frequently reduce the chances of producing a successful *P. destructans* culture. To address this, it is recommended to incubate the sample at temperatures between 7 to 10 °C in order to reduce the growth of other cultivable agents co-occurring in the sample [42]. Likewise, use of an antibiotic supplement (dosage and type may vary) in the culture media is highly recommended. At present, there is still a need for selective media that allow isolation of the fungus from environmental samples containing either *P. destructans* mycelia or spores (i.e. conidia)*,* or both together [52].

The bat’s body surface is constantly exposed to microorganisms present in the environment [53]. As with other vertebrates, bats harbour diverse skin microorganisms, several of which may be potential pathogens. If the skin is healthy, the microbiome contributes to host fitness by occupying pathogen adhesion sites to inhibit infectious agents [53, 54]. Indeed, coevolution of vertebrates with their commensal skin microbiota can affect numerous physiological functions, including protection against infections and immune system reaction patterns [55, 56]. Moreover, some bacterial properties suggest an alliance with the host to keep other potential pathogens at bay [57]. Interactions between commensal bacteria and the pathogen on the host’s skin could provide protection against wing membrane damage and decrease the severity of WNS in bats exposed to the fungal agent [58]. Hoyt et al. [29], when studying interactions between bacteria and the WNS fungus on bat skin, noted that pseudomonads naturally occurring on bats inhibited the growth of *P*. *destructans *in vitro*.* Pseudomonads are ubiquitous in the environment and are known to have antifungal properties. While distribution of *Pseudomonas fluorescens* varies seasonally, recovery tends to be highest in spring and lower in winter [59], which is consistent with this bacterial species being psychrophilic [60, 61]. When testing interactions between fungal and bacterial isolates, all *Pseudomonas* isolates were able to inhibit the *Pseudogymnoascus* fungus [62], while *Serratia* isolates mostly did not [29]. Interestingly, *Serratia* isolates produce mainly keratinases and collagenases [63, 64], suggesting that *Serratia* may hypothetically “prepare” the skin for the fungus. These bacteria are also known to produce prodigiosines [65], which are reported to have antibacterial, immunosuppressive and cellular apoptosis-inducing properties, which might further deteriorate the skin condition. Our results showed no statistical difference in the success of *P. destructans* cultivation from wing membrane biopsies in the presence of *Serratia* spp. and/or *Pseudomonas* spp. despite the relatively higher percentage of *Pd* isolates obtained in the presence of *Serratia* spp. (Fig. 4). This observation may have been due to overall microbial overgrowth in both cases. In addition to physical competition for space, growth of microorganisms can also be limited by the temperature and length of incubation period, medium composition and aerobic conditions [42, 66–68].

It has been shown that the *P. destructans* fungal pathogen is an overproducer of riboflavin [24]. Moreover, it is the photochemical quality of riboflavin [69] and its hyperaccumulation within the infected skin tissue that is responsible for the distinctive orange-yellow fluorescence under UV light, which is used to screen bats for WNS [15]. Riboflavin, and its derivative lumichrome, have been shown to activate the LasR bacterial quorum sensing receptor of *Pseudomonas aeruginosa* [70]. Hypothetically, secretion of these signalling molecules into the extracellular environment could interfere with quorum sensing regulation, trigger population-level density-dependent changes in genes expression of the microbial community associated with bat skin infected with *P. destructans*, or help in establishing a biofilm on the skin during infection [71]. The viability of *P. destructans* may be reduced by host immune-inflammatory responses and, consequently, is thought to be lower in samples collected during the early post-hibernation period [72, 73].

In our experience, the yield of a viable *P. destructans* culture isolate can be improved significantly through adequate sample transport conditions, including an unbroken cold-chain and protection of tissue samples against drying out, and the use of glucose yeast-extract agar with chloramphenicol as the culture medium.

As novel pathogens can seriously impact wildlife, there is a real need to fully understand their biology, pathogenesis and epidemiology. To address this, viable fungal isolates of *P. destructans* are required for epidemiological and phylogeographical studies. If the fungus is not visible on a bat at the time of sample collection from a suspect individual for WNS, then a UV-guided biopsy technique would appear to be the best choice for obtaining a viable *P. destructans* culture. Indeed, UV-guided biopsy sample collection is essential for European and Asian bat species, which only rarely show visible fungal growths, when inspected in their hibernacula. While fungal cultures based on both skin swabs and wing membrane tissue biopsies can be utilised for monitoring and surveillance of *P. destructans* in bat populations, wing membrane biopsies guided by UV light for skin lesions proved higher efficacy.

## Data Availability

All data needed to evaluate the conclusions are present in the paper.
